# Selection of stable reference genes for RT-qPCR in *Rhodococcus opacus* PD630

**DOI:** 10.1038/s41598-018-24486-w

**Published:** 2018-04-16

**Authors:** Drew M. DeLorenzo, Tae Seok Moon

**Affiliations:** Department of Energy, Environmental and Chemical Engineering, Washington University in St. Louis, St. Louis, Missouri 63130 USA

## Abstract

*Rhodococcus opacus* PD630 is a gram-positive bacterium with promising attributes for the conversion of lignin into valuable fuels and chemicals. To develop an organism as a cellular factory, it is necessary to have a deep understanding of its metabolism and any heterologous pathways being expressed. For the purpose of quantifying gene transcription, reverse transcription quantitative PCR (RT-qPCR) is the gold standard due to its sensitivity and reproducibility. However, RT-qPCR requires the use of reference genes whose expression is stable across distinct growth or treatment conditions to normalize the results. Unfortunately, no in-depth analysis of stable reference genes has been conducted in *Rhodococcus*, inhibiting the utilization of RT-qPCR in *R*. *opacus*. In this work, ten candidate reference genes, chosen based on previously collected RNA sequencing data or literature, were examined under four distinct growth conditions using three mathematical programs (BestKeeper, Normfinder, and geNorm). Based on this analysis, the minimum number of reference genes required was found to be two, and two separate pairs of references genes were identified as optimal normalization factors for when ribosomal RNA is either present or depleted. This work represents the first validation of reference genes for *Rhodococcus*, providing a valuable starting point for future research.

## Introduction

*Rhodococcus opacus* PD630 (hereafter *R*. *opacus*) is a gram-positive, oleaginous bacterium that possesses beneficial traits for the conversion of lignin into valuable fuels and chemicals^1–4^. Some of these features include a number of catabolic pathways for lignin-derived aromatic compound consumption and a high native tolerance towards these and other inhibitory lignocellulosic biomass breakdown products^2–5^. Furthermore, *R*. *opacus* can consume multiple types of carbon sources simultaneously^6^, thereby facilitating higher rates of feedstock conversion. Additionally, this organism can direct a large fraction of its cellular resources to the production of biofuel precursors (up to ~78% triacylglycerol [TAG] of cell dry weight)^1^. *R*. *opacus* has been previously engineered to facilitate lignocellulose conversion^3,4,7,8^ and a substantial genetic toolbox has recently been developed^9,10^. However, a deep understanding of this organism’s metabolism and any heterologous pathways being expressed is required to maximize its potential.

A number of technologies exist for examining an organism’s gene expression (i.e. the transcriptome), which is the first step to a systems level understanding. One such technology is the microarray, which allows for gene expression profiling^11^. RNA sequencing (RNA-Seq) is a newer technology that has become the default method for examining the entire transcriptome of an organism^12^. However, it can add additional costs if only several genes are of interest, is limited when mRNA concentrations are low (although this is changing with the advent of single cell sequencing^13^), and generally still requires corroboration via additional quantitative methods^12^. One such complimentary method is reverse transcription quantitative PCR (RT-qPCR), which is considered the gold standard of mRNA quantification due to its high sensitivity, reproducibility, speed, ability to examine numerous samples simultaneously, and large dynamic range^14,15^. Both microarrays and RT-qPCR require the use of an internal standard, optimally a gene that is stably expressed across the tested growth or treatment conditions, to normalize expression data between samples and conditions^16^.

Unfortunately, no in-depth analysis of stable reference genes (RGs) has been performed in *Rhodococcus*, limiting the ability to quantitatively analyze gene expression. One qPCR study in *Rhodococcus equi* even stated that no reference gene was included in their experiment and that such an inclusion could have improved their work^17^. We could find only two examples of reference genes previously reported in *Rhodococcus*. The first reference gene was a gene encoding sigma factor A (*sigA*) in *Rhodococcus* sp. RHA1^18^, although no justification for this choice was provided. The second reference gene was a gene encoding DNA Polymerase IV, which was used in *Rhodococcus* sp. RHA1, *Rhodococcus jostii*, and *Rhodococcus erythropolis*, and this choice was justified based on a microarray experiment performed in *Rhodococcus* sp. RHA1^19,20^. Both of these reference genes were used in isolation and their characterization was incomplete, which fails to satisfy the current minimum information guidelines for publication of quantitative PCR experiments (i.e. MIQE guidelines stating that the minimum number of reference genes needs to be quantitatively determined and that one gene is not generally sufficient for normalization)^21,22^.

In this work, we identified ten candidate reference genes (RGs) and examined the stability of their expression in *R*. *opacus* across four distinct growth conditions using three mathematical models (BestKeeper^23^, NormFinder^24^, and geNorm^16,25^). Additionally, the minimum number of required reference genes was identified. Two different sets of genes were identified as optimal normalization factors (NFs) depending on whether ribosomal RNA (rRNA) is either present or depleted. This work facilitates the utilization of RT-qPCR in *R*. *opacus*, in addition to providing a valuable preliminary set of reference genes for further future validation in other *Rhodococcus* spp.

## Results and Discussion

### Choice of candidate reference genes

Two methods were utilized for the selection of candidate reference genes (RGs). The primary approach used our previously published transcriptomic data collected from *R*. *opacus* grown in a minimal salts medium with either glucose or phenol to identify stably expressed genes^3^. We selected nine genes as candidates (RG1 to RG9; Table 1) whose DeSeq 2 normalized transcript level did not vary significantly between the two growth conditions, whose DeSeq 2 expression value was greater than 750, and whose coding region is at least 350 bp in length^3,26^. The secondary approach utilized a literature review which found that *sigA* had been previously used as a RG in *Rhodococcus* sp. RHA1^18^ and that a DNA Polymerase IV gene has been previously used in *Rhodococcus jostii*, *Rhodococcus erythropolis*, and *Rhodococcus* sp. RHA1 as a RG^19,20^. *sigA* was ruled out due to no justification for its selection as a RG being provided^18^. As PD630_RS27310 is annotated as a DNA Polymerase IV gene in *R*. *opacus*, it was selected as the tenth candidate RG (RG10; Table 1).

**Table 1:** List of candidate reference genes (RGs). The amplicon size, PCR efficiency percentage, minimum and maximum threshold cycle (C_T_) values observed across all tested growth conditions, the standard deviation of C_T_ values across all tested growth conditions (determined by BestKeeper), and the C_T_ value of the no template control (NTC) are listed for each RG. See Supplementary Figure 1 for standard curves used to calculate PCR efficiency. See Supplementary Figure 2 for melting curve analysis. See Supplementary Table 1 for oligonucleotide sequences and melting and annealing temperatures.

Reference gene (RG)	Gene number	Gene annotation	Amplicon size (bp)	PCR efficiency	Min C_T_	Max C_T_	C_T_ Std Dev	NTC C_T_
RG1	PD630_ RS22865	Pup-protein ligase	157	94%	23.44	25.23	0.40	N.d.
RG2	PD630_ RS20570	Hypothetical protein	87	94%	25.86	28.20	0.67	N.d.
RG3	PD630_ RS03840	23S rRNA	87	95%	8.74	10.16	0.33	N.d.
RG4	PD630_ RS15810	Polyribonucleotide nucleotidyltransferase	115	101%	22.93	27.41	1.37	N.d.
RG5	PD630_ RS25785	L,D-transpeptidase Mb0493	90	101%	24.56	25.96	0.37	N.d.
RG6	PD630_ RS13910	NAD(P)H dehydrogenase	82	100%	25.47	29.01	1.13	N.d.
RG7	PD630_ RS25530	ATP-dependent Clp protease ATP-binding subunit ClpX	94	92%	24.93	25.77	0.21	N.d.
RG8	PD630_ RS01395	16S rRNA	101	92%	10.48	11.74	0.29	N.d.
RG9	PD630_ RS37755	rRNA small subunit methyltransferase G	102	92%	28.32	30.33	0.46	N.d.
RG10	PD630_ RS27310	DNA polymerase IV	71	94%	28.34	30.61	0.61	N.d.

N.d. = no amplification detected.

### RT-qPCR primer characterization and data collection

**Figure 1:**
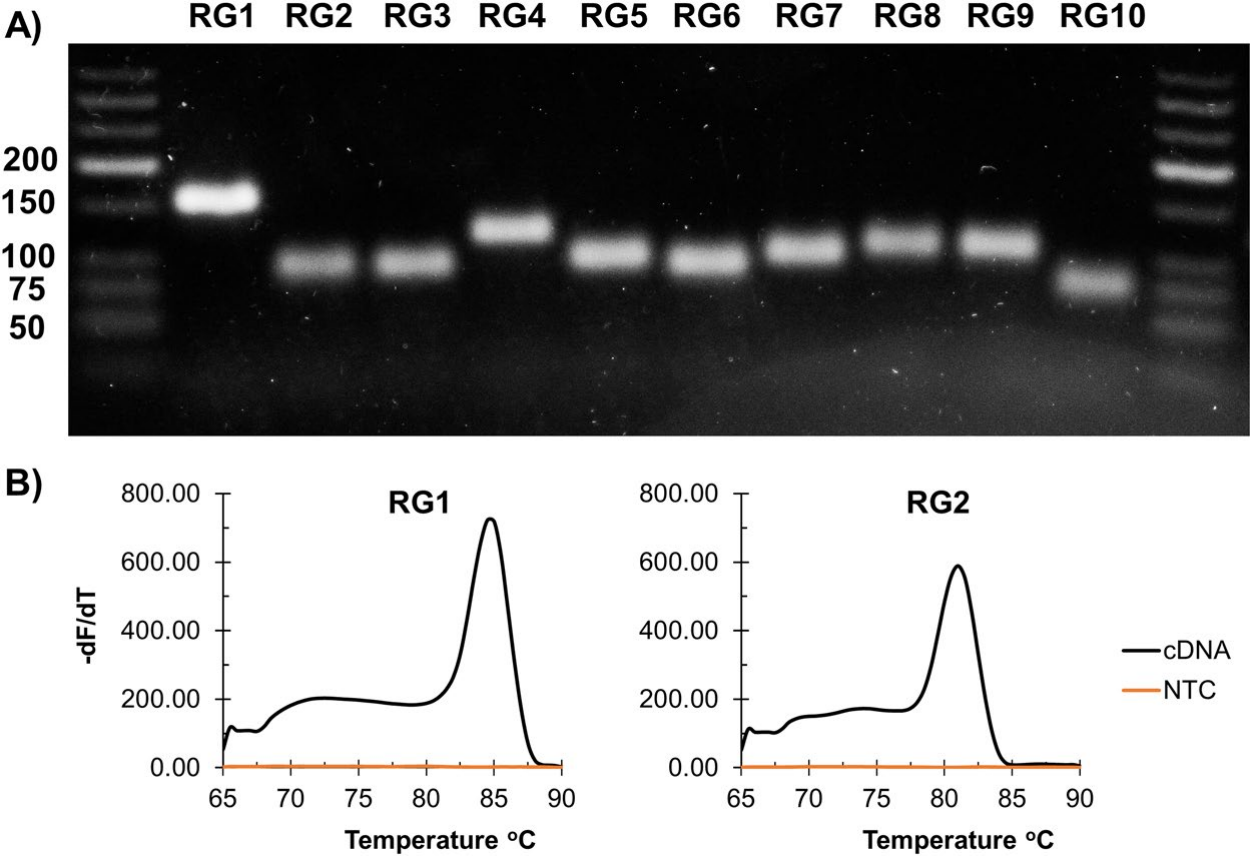
Confirmation of specificity of primers used in RT-qPCR analysis. (**A**) Contrast enhanced image of electrophoresis gel confirming amplicon size and primer specificity using RT-qPCR amplification product. The sizes for nucleotide ladder are indicated to left of bands (50 to 200 bp). The size of each amplicon is denoted in Table 1. The original non-enhanced gel image, in addition to the corresponding gel image of no template controls (NTCs), can be found in Supplementary Figure 5. (**B**) Representative melt curves post RT-qPCR for RG1 and RG2 as well as the corresponding NTCs (see Supplementary Figure 2 for all melt curves).

RT-qPCR primers were designed based on previously published suggestions^22^, in addition to the specific criteria discussed in the Supplementary Materials. The size and specificity of each amplicon was confirmed using agarose gel electrophoresis and melting curve analysis (Fig. 1 and Supplementary Figure 2), and the no template controls (NTCs) demonstrated non-detectable levels of amplification (Table 1 and Supplementary Figure 2). To calculate the PCR amplification efficiency, a ten-fold serial dilution of template DNA (PCR amplified product) was performed and followed by qPCR (Supplementary Figure 1). A linear regression analysis was performed on the resultant C_T_ values to confirm the linearity of each serial dilution and to calculate the PCR amplification efficiency (Table 1 and Supplementary Figure 1). As all serial dilutions had an acceptable R^2^ of at least 0.97 and PCR efficiencies ranged from 92 to 101%, all primer sets were deemed suitable for qPCR (Table 1 and Supplementary Figure 2).

*R*. *opacus* was cultured in four distinct growth conditions, and RNA was extracted from biological triplicate cultures for candidate RG expression stability analysis. The growth conditions are described in full in the Materials and Methods, but in summary they consist of a minimal salts medium with glucose and either high nitrogen (HN) or low nitrogen (LN), a rich tryptic soy broth medium (TSB), or a minimal salts medium with phenol and high nitrogen (PHE). Each medium was selected based on growth conditions that *R*. *opacus* is likely to experience during general research endeavors and based on the diverse predicted changes in metabolic topology required for catabolism of each respective feedstock. Glucose was chosen as a representative sugar feedstock as it is frequently provided in *R*. *opacus* cell cultures^3,9,27^. High and low nitrogen concentrations were examined because *R*. *opacus* undergoes a metabolic flux shift to produce large quantities of industrially relevant lipids during nitrogen deprivation^1,28,29^. TSB was selected as it contains an array of amino acids, which are utilized by a diverse set of metabolic pathways^30^. Phenol was selected as a representative aromatic compound that may be found in depolymerized lignin^3,4^ and because it requires a different subset of metabolic pathways than glucose and amino acids^6^.

**Figure 2:**
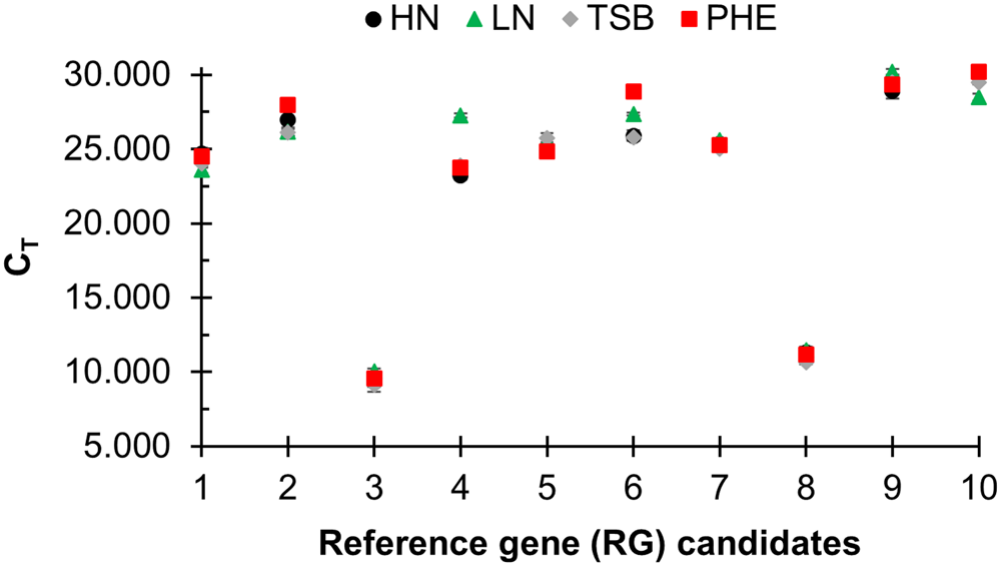
C_T_ values for ten candidate reference genes in *R*. *opacus*. RNA was extracted from biological triplicates of *R*. *opacus* grown in four distinct growth conditions (HN [circle], LN [triangle], TSB [diamond], and PHE [square]; see Materials and Methods for full description). RT-qPCR was performed in technical triplicate on each biological replicate. Each point represents the average threshold cycle (C_T_) value of all replicates for the listed gene and growth condition. Error bars represent one standard deviation.

RT-qPCR was then performed on the cDNA generated from the triplicate RNA samples collected from the HN, LN, TSB, and PHE growth conditions (Fig. 2). The averaged raw C_T_ values ranged from 9.2 to 30.2 for RG1 through RG10 across all growth conditions. The minimum and maximum C_T_ values, in addition to the standard deviation, observed for each candidate RG across all growth conditions are listed in Table 1. A visual appraisal of Fig. 2 can provide initial insight into candidate RG expression stability, as some RGs demonstrated a tight clustering of C_T_ values (e.g. RG1, RG3, RG5, RG7, RG8, and RG9) while others demonstrated a spread in C_T_ values (e.g. RG2, RG4, RG6, and RG10) across growth conditions.

### Expression stability of candidate reference genes across distinct growth conditions

**Figure 3:**
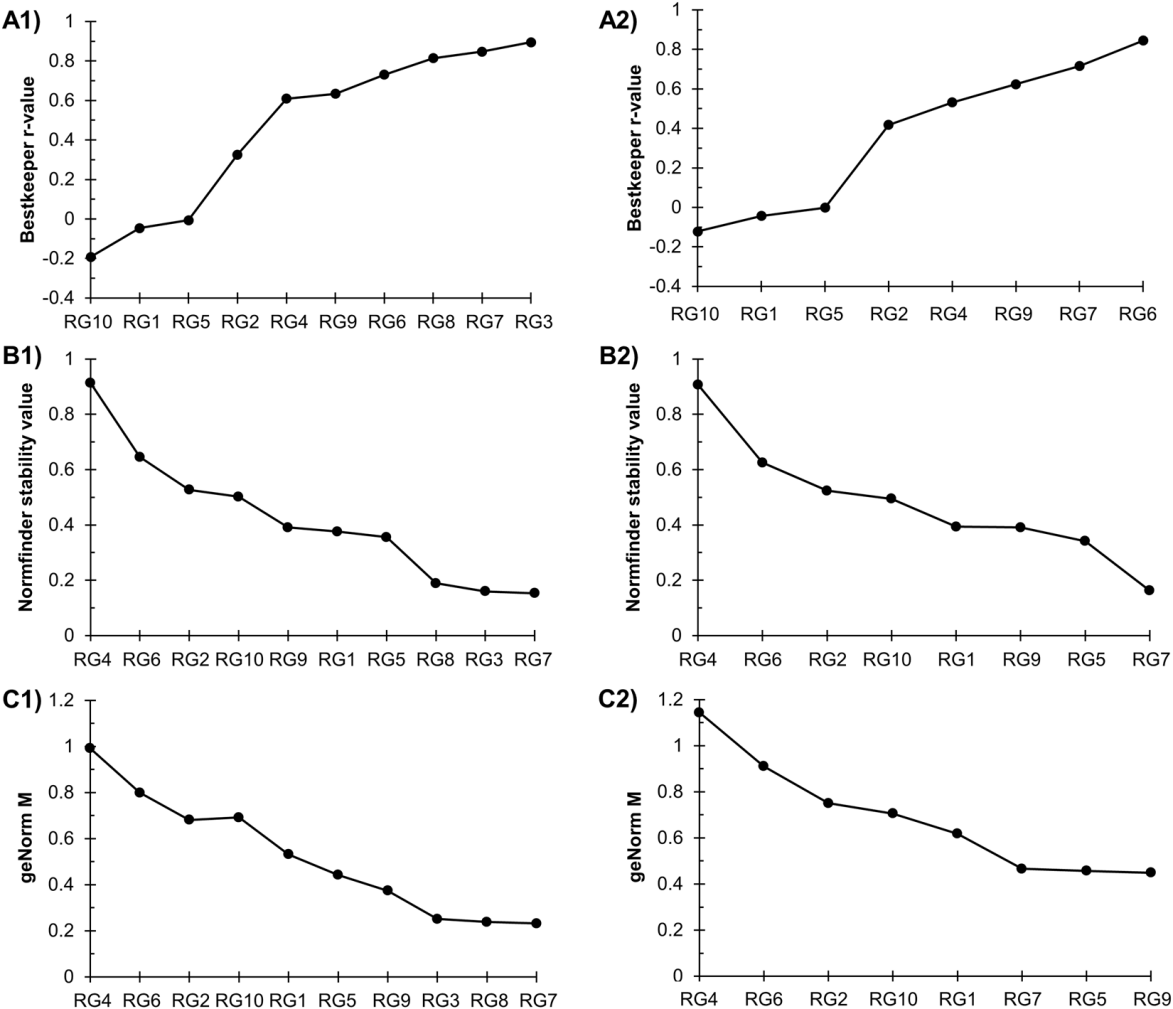
Rankings of candidate reference genes. Genes were ranked from the least stable (on the left) to the most stable (on the right). Analysis was performed after pooling C_T_ data across all four growth conditions. The designation of 1 means that the analyses were performed on all ten candidate RGs. The designation of 2 means that the analyses were performed on the eight non-rRNA candidate RGs. (**A**) Genes were ranked according to their BestKeeper r-value. BestKeeper r-value significance can be found in Supplementary Tables 3 and 4. (**B**) Genes were ranked according to their Normfinder stability value. (**C**) Genes were ranked according to their geNorm M value.

Candidate RG expression stability was quantitatively examined using three different statistical programs: BestKeeper^23^, NormFinder^24^, and geNorm^16,25^. Each of these programs generates a gene expression stability coefficient (r-value for BestKeeper, stability value for Normfinder, and M value for geNorm) and rank order for the candidate RGs (see Materials and Methods for full description; Fig. 3). Additionally, candidate RGs were ranked based on their C_T_ standard deviation calculated by BestKeeper (Supplementary Table 2). A C_T_ standard deviation greater than 1 is considered unstable^23^.

All analyses identified the same three candidate RGs, albeit in different rank orders, as being the most stably expressed across all four growth conditions. According to BestKeeper, the top three most stable RGs from 1^st^ to 3^rd^, based on the smallest standard deviation of C_T_ values, were RG7, RG8, and RG3 (Supplementary Table 2). The top three RGs with the lowest BestKeeper r-value from 1^st^ to 3^rd^ were RG3, RG7, and RG8 (Fig. 3A1). Normfinder determined that the top three most stable RGs from 1^st^ to 3^rd^, based on its stability value calculation, were RG7, RG3, and RG8 (Fig. 3B1). Finally, geNorm determined that the top three most stable RGs from 1^st^ to 3^rd^, based on its M value, were RG7, RG8, and RG3 (Fig. 3C1).

Two of the top three most stable candidate RGs encode rRNAs (RG3 and RG8), which is consistent with previous works in other organisms that identified an rRNA gene as a stable RG^31,32^. However, rRNA is often depleted from mRNA samples being prepared for RNA-Seq^33^. If RT-qPCR is to be used to corroborate RNA-Seq results, other non-rRNA RGs need to be identified. All analyses were repeated on the non-rRNA candidate RGs, and two genes (RG7 and RG9) appeared in the top 3 rankings of three analyses, while another (RG5) appeared in the top 3 rankings of two analyses (Fig. 3). The BestKeeper r-value identified the top three non-rRNA RGs from 1^st^ to 3^rd^ as RG6, RG7, and RG9 (Fig. 3A2). As RG6 had a C_T_ standard deviation greater than 1 (Supplementary Table 2), it was ruled out as a candidate. Normfinder identified the top three non-rRNA RGs from 1^st^ to 3^rd^ as RG7, RG5, and RG9 (Fig. 3B2). geNorm identified the top three non-rRNA RGs from 1^st^ to 3^rd^ as RG9, RG5, and RG7 (Fig. 3C2).

### Minimum required number of reference genes

**Figure 4:**
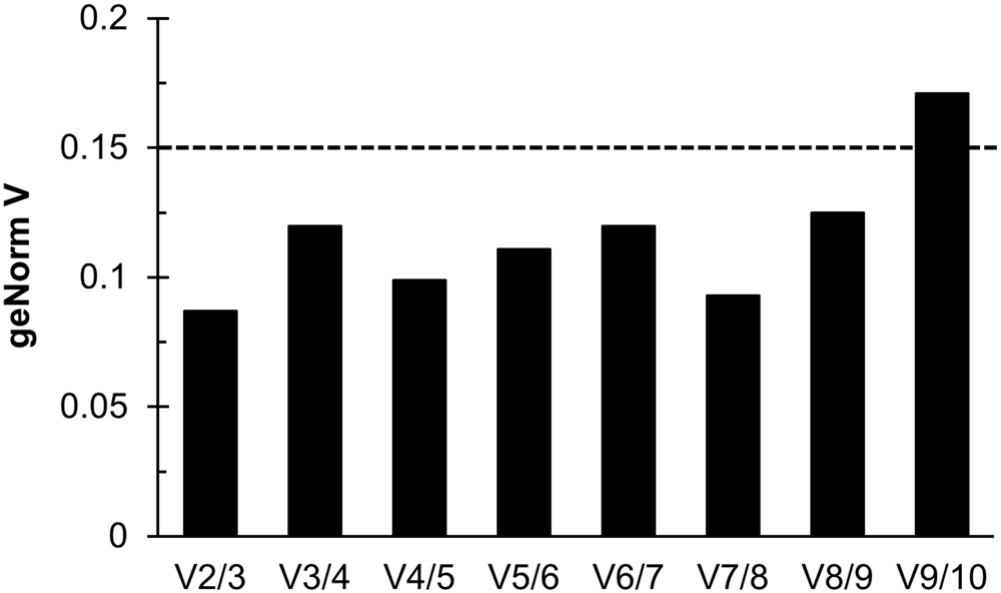
Minimum number of reference genes. The pair-wise variation Vn/Vn + 1, where n represents the number of RGs used in the normalization factor (NF), was calculated by geNorm to determine the minimum number of RGs required for normalization. A geNorm V value below 0.15 signifies that no additional benefit is gained from increasing the number of reference genes from n to n + 1. The dashed line represents a V value of 0.15.

Analyses of candidate RG expression stability identified several consistently transcribed genes across the four distinct growth conditions, but it was still unknown how many RGs were required for optimal normalization of expression data. The geNorm software is also capable of producing a V value (see Materials and Methods), which produces a quantitative suggestion for the ideal number of required RGs. By calculating the pairwise variation of V_n_/V_n+1_ (V value), where n is the number of RGs, the benefit of using n versus n + 1 RGs for normalization can be quantified. A V value below 0.15 signifies that there is no additional benefit from adding another RG to the normalization factor (NF)^16,25^. A V value was calculated for all ten candidate RGs and the non-rRNA RG subset, and both had a V_2_/V_3_ value below 0.15 (0.087 and 0.145, respectively), meaning that there is no benefit of using three RGs over two RGs in the NF (Fig. 4 and Supplementary Figure 3).

### Validation of the selected reference genes

**Figure 5:**
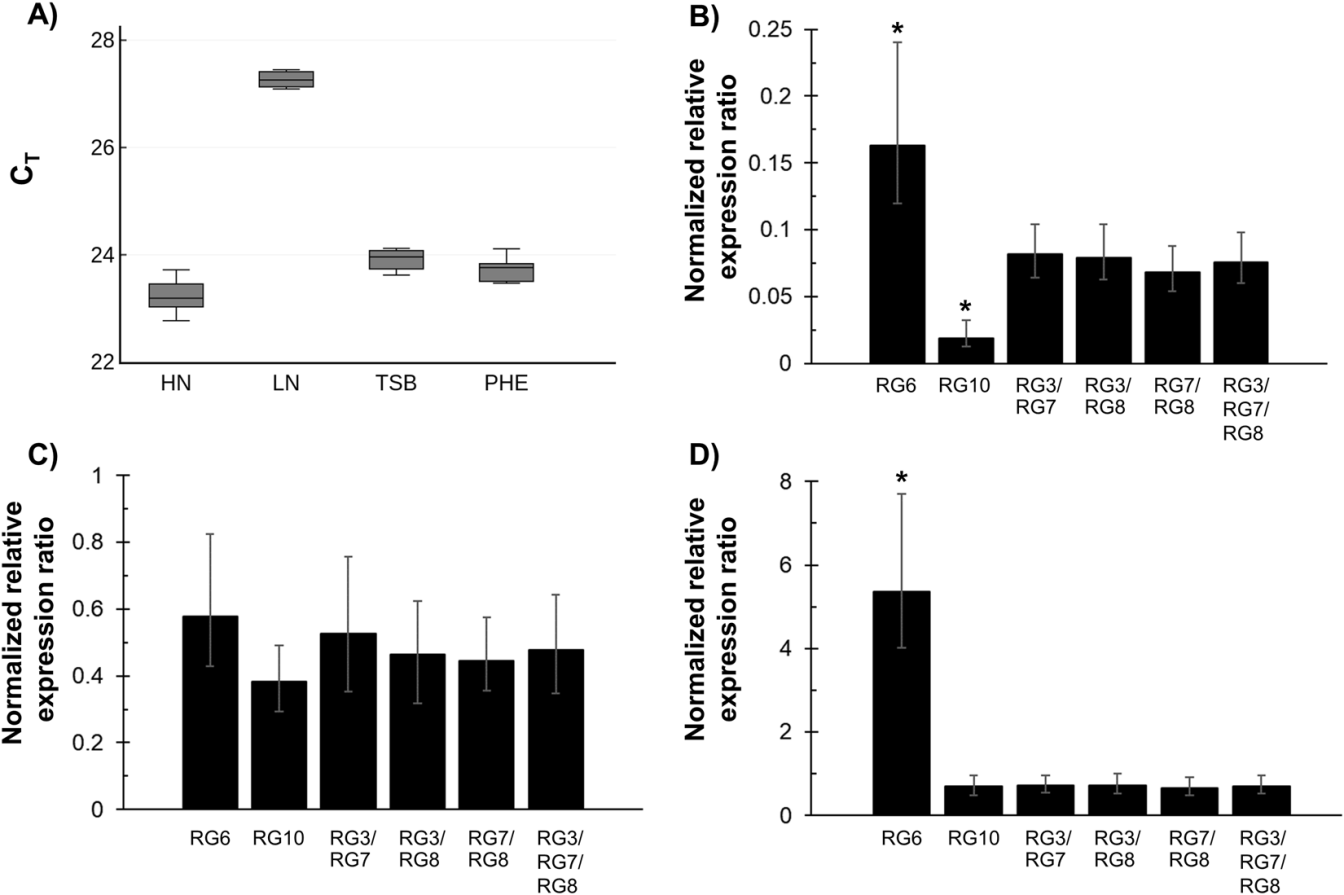
Effect of reference gene choice on RT-qPCR normalization. (**A**) Box plots of averaged PD630_RS15810 expression data (C_T_) for all four growth conditions (HN, LN, TSB, and PHE). Each gray box represents the first through third quartiles, the solid black line represents the median, and the whiskers represent the minimum and maximum values. (**B**–**D**) The normalized relative expression ratio of PD630_LPD05540 going from HN to either LN (**B**), TSB (**C**), or PHE (**D**). The expression data was normalized with a NF, including RG6, RG10, RG3/RG7, RG3/RG8, RG7/RG8, and RG3/RG7/RG8 using REST 2009. Error bars represent the 95% confidence interval (CI) as calculated by REST 2009. Stars indicate that a 95% CI range falls outside of the 95% CI range of the RG3/RG7/RG8 (and RG7/RG8) normalized ratio.

To examine the effect of utilizing different combinations of RGs in the NF, a normalized expression analysis was performed using expression data from PD630_RS15810 (RG4) grown under the four distinct growth conditions (Fig. 5). A box plot visualizing the non-normalized expression data for PD630_RS15810 was generated to show the general trends in expression level changes across the four growth conditions (Fig. 5A). The normalized relative fold changes in expression going from the HN condition to either the LN, TSB, or PHE condition were calculated using REST 2009 with one of six NFs, including RG6, RG10, RG3/RG7, RG3/RG8, RG7/RG8, and RG3/RG7/RG8 (Fig. 5). Confidence intervals of 95% were calculated by REST 2009 and used for comparison between different NFs. RG6 was chosen as an example of a poor RG candidate, while RG10 was chosen as DNA Polymerase IV had been used as a RG in other *Rhodococcus* spp.^19,20^. All two-component combinations (NF_2_) of RG3, RG7, and RG8 were examined as it was unclear whether there were differences between them, in addition to a NF comprising all three to confirm that there was no significant difference between a NF_2_ and a NF_3_.

The results of the normalized expression analysis revealed that there was no significant difference in the normalized relative fold change ratio going from the HN condition to either the LN, TSB, or PHE condition when using a NF_2_ comprising any two selections of RG3, RG7, and RG8 or a NF_3_ comprising all three genes. As the NF_2_ comprising RG7 and RG8 (RG7/RG8) had the smallest 95% confidence interval, it was chosen as the optimal pair of RGs for future use in *R*. *opacus* under the tested growth conditions. RG7/RG8 normalization revealed that PD630_RS15810 expression with 95% confidence was downregulated between 0.054 to 0.088-fold when going from HN to LN, 0.355 to 0.575-fold when going from HN to TSB, and 0.494 to 0.916-fold when going from HN to PHE (Fig. 5).

The effect of non-optimal RGs was also examined in comparison to the NF_3_ comprising RG3, RG7 and RG8. When PD630_RS15810 expression was normalized with RG6, there was a significantly different 95% confidence range (p < 0.05) of 0.120 to 0.240-fold downregulation when going from HN to LN, a comparable 0.430 to 0.824-fold downregulation going from HN to TSB, and a significantly different (p < 0.05) 4.009 to 7.703-fold upregulation when going from HN to PHE (Fig. 5). When PD630_RS15810 expression was normalized with the literature-based selection RG10, there was a significantly different 95% confidence range (p < 0.05) of 0.013 to 0.032-fold downregulation when going from HN to LN, a comparable 0.294 to 0.490-fold downregulation going from HN to TSB, and a comparable 0.480 to 0.971-fold downregulation when going from HN to PHE. These results demonstrate that a poorly selected RG can substantially alter the observed change in gene expression (e.g. RG6), and that RGs should be examined in all relevant growth conditions prior to use as they may be stable in some instances but not in others (e.g. RG10).

To determine an optimal NF for a scenario where rRNA has been depleted, a normalized expression analysis was performed again on PD630_RS15810 using the top three non-rRNA candidate RGs (RG5, RG7, and RG9). The normalized expression ratio of all NF_2_ combinations of these three genes, in addition the NF_3_ comprising all of them, was examined in comparison to the best NF_2_ comprising RG7 and RG8 (Supplementary Figure 4). The NFs including RG5/ RG7, RG5/RG9, and RG5/RG7/RG9 all produced 95% confidence normalized expression fold change ranges that were significantly different (p < 0.05), compared to the expression fold change range produced by RG7/RG8 when going from HN to TSB (Supplementary Figure 4C). The NF comprising RG7 and RG9 was comparable to RG7/RG8 in all scenarios, with 95% confidence normalized expression ranges of PD630_RS15810 similarly changing 0.072 to 0.127-fold when going from HN to LN, 0.534 to 0.909-fold when going from HN to TSB, and 0.550 to 1.096-fold when going from HN to PHE.

## Conclusion

This study represents the first in-depth analysis of stable reference genes in *Rhodococcus* and identified two comparable normalization factors in *R*. *opacus* for samples with or without rRNA. The expression of ten candidate reference genes was examined across four distinct growth conditions (HN, LN, TSB, and PHE) using three mathematical models (BestKeeper, Normfinder, and geNorm) to identify and rank gene expression stability. Based on the geNorm V value and corroboration via a normalized gene expression analysis using REST 2009, only two reference genes are required for an optimal normalization factor in *R*. *opacus*. For samples containing rRNA, the two best reference genes were RG7 (PD630_RS25530; ATP-dependent Clp protease ATP-binding subunit ClpX) and RG8 (PD630_RS01395; 16S rRNA). For samples depleted in rRNA, the best two reference genes were RG7 and RG9 (PD630_RS37755; rRNA small subunit methyltransferase G). The diversity of growth conditions tested in this work bestows confidence in our selections of reference genes for *R*. *opacus*, in addition to providing a valuable starting point in the choice of reference genes when studying other *Rhodococcus* spp.

## Materials and Methods

### Strain and culture conditions

*Rhodococcus opacus* PD630 (DSMZ 44193) was grown in either a minimal salts medium as previously described (minimal media recipe B)^9^, with one of three distinct combinations of carbon and nitrogen sources (discussed in detail in the Gene expression studies section), or a rich tryptic soy broth (TSB) medium. Cultures were incubated at 30 °C and 250 rotations per minute (rpm). For the growth experiment, all cultures were grown in triplicate 10 mL cultures with an initial optical density at 600 nm (OD_600_) of 0.2. Chemicals were purchased from Sigma-Aldrich (St. Louis, MO) unless otherwise noted. MIQE guidelines were applied as appropriate to the design and execution of experiments^21,22^.

### Candidate reference gene selection and primer design

The selection of candidate reference genes was based on either previously published transcriptomic data for *R*. *opacus*^3^ or literature for other *Rhodococcus* spp.^19,20^. RG1 through RG9 were selected as potentially stable genes based on analysis of RNA-Seq data performed on *R*. *opacus* grown in a minimal salts medium with either glucose or phenol^3^. RG10 was chosen based on its gene annotation as DNA Polymerase IV, as other *Rhodococcus* spp. studies have used a DNA Polymerase IV gene as a reference gene^19,20^. RT-qPCR primers (Integrated DNA Technologies) were designed based on literature suggestions^21,22^ and guidelines discussed in the Supplementary Materials.

### Primer amplification efficiency and specificity

To calculate primer amplification efficiency, a serial dilution of template DNA and a linear regression analysis of the resultant qPCR results were required. PCR product was obtained by using 0.5 μL of GoTaq G2 polymerase (Promega), 10 μL of GoTaq buffer, 1.5 μL of a mixture containing the forward and reverse primers (5 μM each), 0.5 μL of genomic DNA (gDNA), 1 μL of 10 mM dNTPs (GBiosciences), and 36.5 μL of H_2_O; and thermocycler conditions of 95 °C for 2 min, followed by 35 cycles of 95 °C for 15 s, 62 °C for 30 s, and 72 °C for 30 s. PCR products were gel extracted using the Zymoclean DNA Gel Recovery kit (Zymo Research) and prepared for qPCR using the DNA Clean and Concentrator kit (Zymo Research). Five rounds of a 10-fold serial dilution were performed on the purified PCR product. qPCR was performed in duplicate on the serially diluted PCR product using a Bio-Rad CFX96 real time thermocycler, TempPlate 96-well semi-skirt 0.1 mL PCR plates (USA Scientific), and Power SYBR Green PCR Master Mix (Applied Biosystems). 10 μL of Power SYBR Green PCR Master Mix, 0.5 μL of a mixture containing the forward and reverse primers (5 μM each), 1 μL of PCR product, and 8.5 μL of H_2_O were used for each reaction. Cycling conditions were 95 °C for 10 min, followed by 40 cycles of 95 °C for 15 s, 62 °C for 20 s, 72 °C for 20 s, and a fluorescence measurement. A fluorescence threshold value of 750 was set for all reactions. To confirm that no off-target amplification had occurred, a melting curve analysis was performed at the end of each qPCR program and a single melting peak was verified for each amplicon. A linear regression was performed on the threshold cycle (C_T_) values observed from each serial dilution to confirm linearity (R^2^) and to calculate the equation of the line, which was used to estimate the efficiency of the PCR (Equation 1; Supplementary Figure 1).

1 $${\boldsymbol{PCR}}\,{\boldsymbol{Efficiency}}\,{\boldsymbol{Percentage}}={\bf{100}}\,\ast \,([{\bf{1}}{{\bf{0}}}^{\frac{-1}{{\boldsymbol{standard}}{\boldsymbol{curve}}{\boldsymbol{slope}}}}]-1)$$

### Gene expression studies

To examine gene expression stability, *R*. *opacus* was grown in four distinct growth conditions. The first condition was a rich TSB medium. The other three conditions were minimal salts media with carbon and nitrogen sources as follows: glucose with high nitrogen (HN; 2 g/L glucose and 1 g/L ammonium sulfate), glucose with low nitrogen (LN; 2 g/L glucose and 0.05 g/L ammonium sulfate), and phenol with high nitrogen (PHE; 0.75 g/L phenol + 1 g/L ammonium sulfate). A 5 mL seed culture in a 50 mL glass tube containing the minimal salts medium with 2 g/L glucose and 1 g/L ammonium sulfate was used for the HN, LN, and TSB growth conditions, while the PHE seed culture also had 0.3 g/L phenol added to acclimate the cells to the inhibitory aromatic. To remove remaining glucose and ammonium sulfate from the seed cultures, samples were centrifuged at 3500 relative centrifugal force (rcf), washed with minimal media containing no carbon or nitrogen sources, centrifuged again at 3500 rcf, and re-suspended in their final growth condition media. When the cultures reached mid-exponential phase, the cells were centrifuged at 3500 rcf and re-suspended in DNA/RNA Shield (Zymo Research), per the instructions. Samples were stored at −80 °C until needed for further use.

### RNA extraction and cDNA synthesis

RNA was extracted from the triplicate biological samples stored at −80 °C in the DNA/RNA Shield using the Quick-RNA MiniPrep Plus kit (Zymo Research), per the instructions. The optional ZR BashingBead Lysis Tubes (0.1 and 0.5 mm beads; Zymo Research) were used to lyse the cells. All samples were treated with the TURBO DNA-free kit (Ambion) to remove any gDNA present in the samples and then purified using the RNA Clean and Concentrator kit (Zymo Research). To confirm that all gDNA was depleted, PCR was performed by using the previously described GoTaq protocol with primers targeting the genome. Gel electrophoresis was performed to confirm that no PCR amplification bands existed. Any samples with persisting gDNA were re-treated with the TURBO DNA-free kit, purified again, and examined via PCR and gel electrophoresis. RNA concentration and purity were quantified using a NanoDrop 2000c (all samples had a 260 nm/280 nm absorbance ratio of 2 to 2.1). 1 ug of RNA per sample was converted to cDNA using the AffinityScript QPCR cDNA synthesis kit (Agilent Technologies).

### RT-qPCR

RT-qPCR was performed in technical triplicate on the biological triplicate RNA extracts using a Bio-Rad CFX96 and Power SYBR Green PCR Master Mix (Applied Biosystems). 10 μL of Power SYBR Green PCR Master Mix, 0.5 μL of a mixture containing the forward and reverse primers (5 μM each), 1 μL of cDNA, and 8.5 μL of H_2_O were used for each reaction. Cycling conditions were 95 °C for 10 min, followed by 35 cycles of 95 °C for 15 s, 62 °C for 20 s, 72 °C for 20 s, and a fluorescence measurement. A threshold value of 750 was set for all reactions. To confirm that no off-target amplification had occurred, a melting curve analysis was performed at the end of each qPCR program and a single melting peak was verified for each amplicon (Supplementary Figure 2). Additionally, a negative control (no template control; NTC) was included for each primer pair (Table 1 and Supplementary Figure 2). Gel electrophoresis was also performed post RT-qPCR to confirm the existence of a single amplicon (Fig. 1A and Supplementary Figure 5). Gel images were captured using a DigiDoc-It imaging system and its accompanying Doc-ItLS software. The original Fig. 1A gel image was cropped, and contrast and exposure settings for the whole image were modified using Adobe Lightroom to improve image interpretation. The original image can be found as Supplementary Figure 5.

### Reference gene expression stability analysis

The expression level stability of the ten candidate RGs across the four distinct growth conditions was assessed and ranked using three commonly used software tools: BestKeeper^23^, NormFinder version 0.953^24^, and geNorm (built into qBase + [Biogazelle])^16,25^ (Figs 3 and 4; Supplementary Tables 2 and 3). Additionally, all analyses were performed again using just candidate RGs 1, 2, 4–7, 9, and 10 to accommodate a situation where rRNA (RG3 and 8) has been depleted from the sample (Fig. 3, Supplementary Figures 3 and 4, and Supplementary Table 4).

BestKeeper is a Microsoft Excel based tool that calculates the standard deviation and coefficient of variance of the C_T_ value for each candidate RG, and creates an index based on the geometric mean of the C_T_ values, which it uses to facilitate pair-wise comparisons against and between RGs. A Pearson correlation coefficient (r-value) is calculated for each RG, with higher r-values representing greater stability (max value of 1)^23^. The technical triplicate C_T_ values for each biological replicate were averaged prior to entry into BestKeeper and all four growth conditions were pooled and analyzed together. Candidate RGs were ranked on both their C_T_ value standard deviation (values > 1.0 deemed unstable^23^; Supplementary Table 2) and their r-value (Fig. 3A1 and A2, and Supplementary Tables 3 and 4).

Normfinder is also a Microsoft Excel based tool that utilizes a model-based approach to compare intra- and inter-group variation between candidate RGs and then generates a stability value and ranking for each candidate gene^24^. The lower the stability value, the more stable the gene’s expression. The technical triplicate C_T_ values for each biological replicate were averaged and samples from all four growth conditions were pooled. Values were then converted to a log-scale per Equation 2, where E is the amplicon specific PCR efficiency, prior to entry into Normfinder^24^. Candidate RGs were ranked on their stability value (Fig. 3B1 and B2).

2 $${\boldsymbol{Log}}\,{\boldsymbol{transformed}}\,{\boldsymbol{value}}={\boldsymbol{E}}{}^{-{{\boldsymbol{C}}}_{{\boldsymbol{T}}{\boldsymbol{avg}}}}$$

geNorm, formerly a Microsoft Excel based tool that has been incorporated into the qBase + software package (Biogazelle), examines the pairwise variation between RGs and creates a stability M value, where lower values represent more stably expressed genes^16,25^. The program iterates its calculations wherein it eliminates the gene with the highest M value and then recalculates the stability of the remaining genes. Thus, geNorm creates a ranking of RG stability (Fig. 3C1 and C2). A geNorm M value lower than 1 is generally considered stable for heterogeneous growth conditions^22^. geNorm can also determine the minimum number of RGs required for optimal RT-qPCR normalization. Various normalization factors (NF) are calculated by first taking the geometric mean of the two most stable RGs’ C_T_ values and then generating additional factors by adding the next best RG until all RGs are used. A pairwise variation (V_n_/V_n+1_) V value is then calculated by comparing the benefit of going from NF_n_ to NF_n+1_, where n is a number of RGs (Fig. 4 and Supplementary Figure 3). Once the V value is less than 0.15, there is no additional benefit of going from n to n + 1^16,25^.

### Validation of reference gene selection

The effect of different combinations of the top candidate RGs was examined by creating multiple different NFs and normalizing the same set of expression data using REST 2009 (Qiagen). The relative expression ratio data of PD630_RS15810 going from the HN condition to either the LN, TSB, or PHE condition was normalized by a NF_2_ comprising any two of the top three RGs identified across all analyses (RG3, RG7, and RG8), in addition to a NF_3_ comprising all three. Additionally, RG6 was used as an example of an unsuitable RG, while RG10 was used based on literature references for other *Rhodococcus* spp^19,20^. 95% confidence intervals were generated by REST 2009. If 95% confidence intervals failed to overlap, they were labelled as significantly different (p < 0.05). As two of the top three RGs identified encode rRNAs (RG3 and RG8), which are frequently depleted when performing RNA-Seq, additional NFs comprising non-rRNA RGs (RG5, RG7, and RG9) were examined in comparison to the overall top NF combination (RG7 and RG8; Supplementary Figure 4).

### Data availability

The datasets generated during the current study are either included in the manuscript or Supplementary Materials or are available from the corresponding author on reasonable request.

## Electronic supplementary material

Supplementary Materials
