# Supplementary material for: Selection of stable reference genes for RT-qPCR in Rhodococcus opacus PD630
Source: Sci Rep. 2018 Apr 16;8:6019. doi: 10.1038/s41598-018-24486-w (PMC5902447; doi:10.1038/s41598-018-24486-w)
Supplement: Supplementary file 1 — Supplementary Materials [file 41598_2018_24486_MOESM1_ESM.pdf]

Supplementary Materials

Selection of stable reference genes for RT-qPCR in *Rhodococcus opacus* PD630

Drew M. DeLorenzo and Tae Seok Moon

Department of Energy, Environmental and Chemical Engineering, Washington University in St. Louis, St. Louis, MO 63130, USA

Supplementary Table 1. Candidate reference gene (RG) primer sequences. The forward and reverse oligonucleotide sequences used for RT-qPCR for each respective RG candidate. Also listed are the predicted melting temperature (T_M) for each respective oligonucleotide and the annealing temperature (T_A) for each oligonucleotide pair as predicted by the ThermoFisher's Tm calculator (https://www.thermofisher.com/us/en/home/brands/thermo-scientific/molecular-biology/molecular-biology-learning-center/molecular-biology-resource-library/thermo-scientific-web-tools/tm-calculator.html). See "RT-qPCR primer design criteria" for how oligonucleotide sequences were selected.

**Reference gene (RG)**	**Primer sequence**	T_M	T_A
RG1	F: CCCGCCGAGTCCGTGTTGTTCTTG<br>R: CGACAGCCGAGTGCGACAACCTCATC	65 ^{O}C<br>67 ^{O}C	60 ^{O}C
RG2	F: CCTGACCTGTCGGCACGAATGAGC<br>R: CCTTCACGGCATCCCCAACGC	65 ^{O}C<br>65 ^{O}C	60 ^{O}C
RG3	F: ATTCCGTGAGTAGTGGCGAGCGAAAGC<br>R: CCACAACCCCACGAATGCAACACCTG	66 ^{O}C<br>65 ^{O}C	60 ^{O}C
RG4	F: GCTGTCGGCTGAGGTCGCCATC<br>R: CTGCTGCGCCATCTTCACCATGTCG	66 ^{O}C<br>65 ^{O}C	60 ^{O}C
RG5	F: CTCACCTTCCGACGTGACGCTGTC<br>R: GCGACGATTGTGGCGGCATCACTC	65 ^{O}C<br>66 ^{O}C	60 ^{O}C
RG6	F: GCCCACGCACCTCGTCGTCG<br>R: GACCTTGACGCCCATCTCGGTGTAGG	67 ^{O}C<br>66 ^{O}C	61 ^{O}C
RG7	F: GCGTCCGTGGTGAACTCCAACTCC<br>R: TCCAGATCCTCTCCGAGCCGAAGAAC	65 ^{O}C<br>65 ^{O}C	60 ^{O}C
RG8	F: CTCGGAGGAAGGTGGGGACGACG<br>R: CCTCACGGTATCGCAGCCCTCTG	65 ^{O}C<br>65 ^{O}C	60 ^{O}C
RG9	F: TGGATAAGCGGCAGCGACCACTTGG<br>R: GGAGTCGGGTGTGGTGAAGGAAGC	66 ^{O}C<br>65 ^{O}C	60 ^{O}C
RG10	F: CGTCGTCGGTGGGTGTTGCATGTC<br>R: TCAGGATGCCGTAGCACCTCGACTG	65 ^{O}C<br>65 ^{O}C	60 ^{O}C

Supplementary Table 2. Ranking of candidate reference genes (RGs) by C_T standard deviation. The ten candidate RGs were ranked by the standard deviation of their C_T values as calculated by Bestkeeper. Analysis was performed on the pooled data set containing all four growth conditions. A standard deviation greater than 1 is considered unstable. The minimum and maximum C_T values for each RG are also provided.

**Rank**	**10**	**9**	**8**	**7**	**6**	**5**	**4**	**3**	**2**	**1**
	RG4	RG6	RG2	RG10	RG9	RG1	RG5	RG3	RG8	RG7
**min [C_T]**	22.93	25.47	25.86	28.34	28.32	23.44	24.56	8.74	10.48	24.93
**max [C_T]**	27.41	29.01	28.20	30.61	30.33	25.23	25.96	10.16	11.74	25.77
**std dev [± C_T]**	1.37	1.13	0.67	0.61	0.46	0.40	0.37	0.33	0.29	0.21

Supplementary Table 3. Bestkeeper ranking with significance value. The ten candidate RGs were ranked by their Bestkeeper r-value calculated on the pooled data set containing the results from all four growth conditions in biological triplicates. Three technical replicates for each biological replicate were averaged prior to input into Bestkeeper. Bolded values represent a p-value < 0.05.

**Rank**	**10**	**9**	**8**	**7**	**6**	**5**	**4**	**3**	**2**	**1**
**Gene**	RG10	RG1	RG5	RG2	RG4	RG9	RG6	RG8	RG7	RG3
**r-value**	-0.192	-0.046	-0.006	0.326	**0.609**	**0.634**	**0.731**	**0.814**	**0.847**	**0.895**

Supplementary Table 4. Bestkeeper ranking with significance value with rRNA candidates removed. The eight candidate RGs were ranked by their Bestkeeper r-value calculated on the pooled data set containing the results from all four growth conditions in biological triplicates. Three technical replicates for each biological replicate were averaged prior to input into Bestkeeper. Bolded values represent a p-value < 0.05.

**Rank**	**8**	**7**	**6**	**5**	**4**	**3**	**2**	**1**
**Gene**	RG10	RG1	RG5	RG2	RG4	RG9	RG7	RG6
**r-value**	-0.122	-0.043	-0.001	0.418	0.532	**0.624**	**0.716**	**0.845**

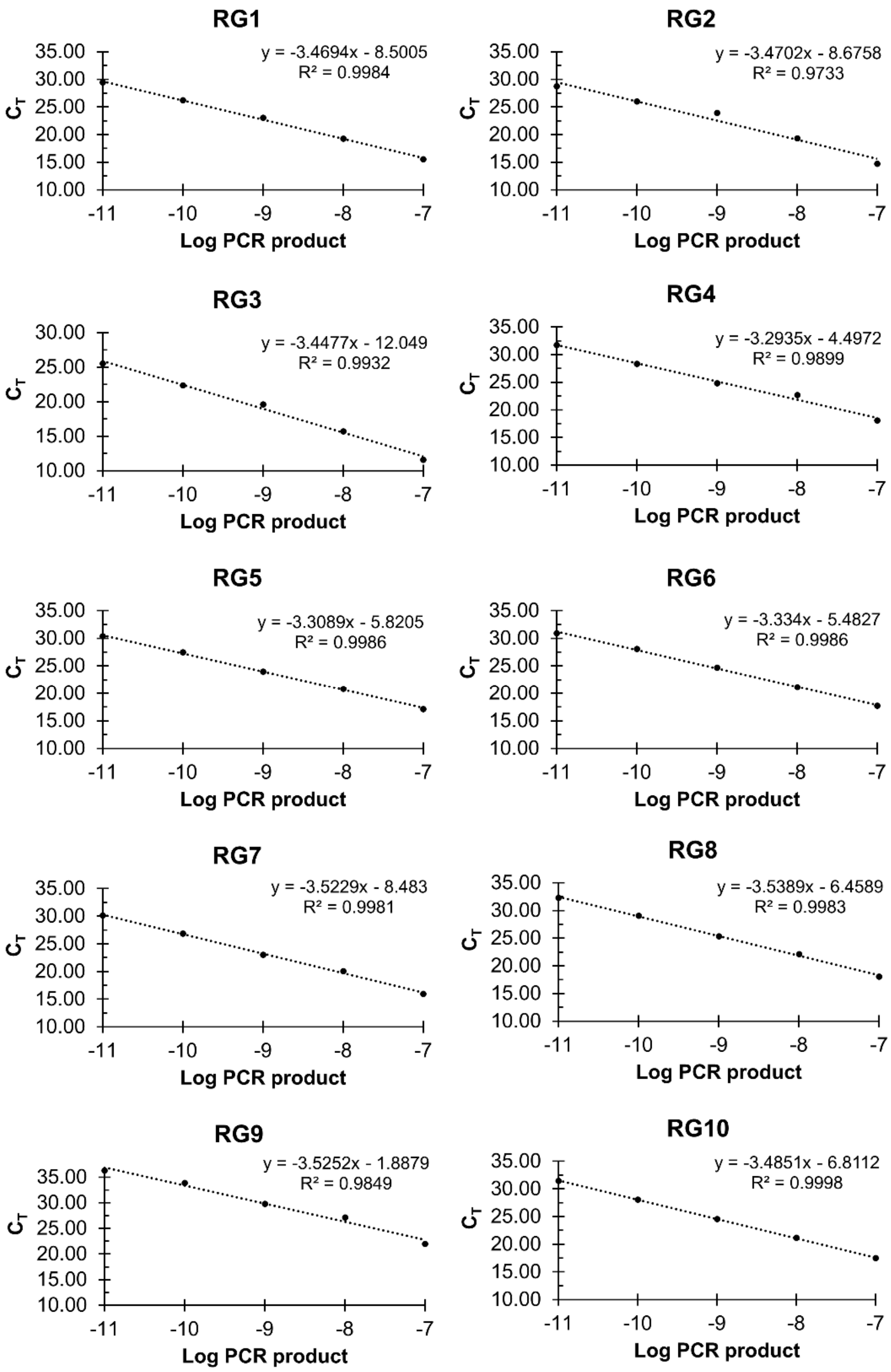

Supplementary Figure 1. Standard curves for RT-qPCR primers. A five-round 10-fold serial dilution was performed on purified PCR product for each RT-qPCR primer pair. Each data point represents the average of duplicate values. A linear regression (dashed line) was performed for each data set, and the equation of the line and R^2 value are listed on each respective plot.

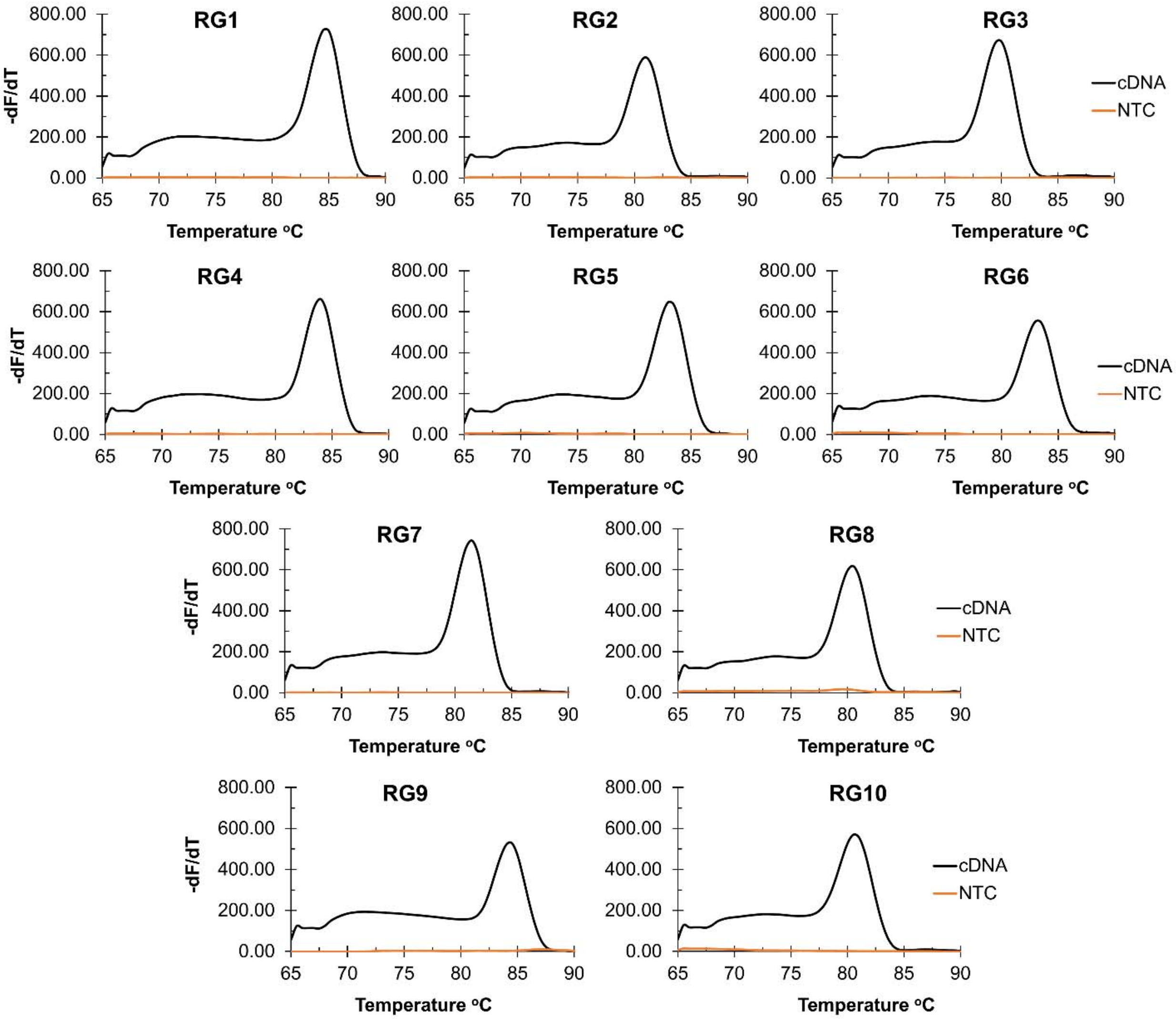

Supplementary Figure 2. Melt curves for candidate reference gene amplicons. A melting curve analysis was performed on samples using the BioRad CFX96 after RT-qPCR to confirm a single amplicon was produced. The cDNA condition (black line) had cDNA added to the PCR reaction, while the NTC (no template control; orange line) condition had H_2O added instead.

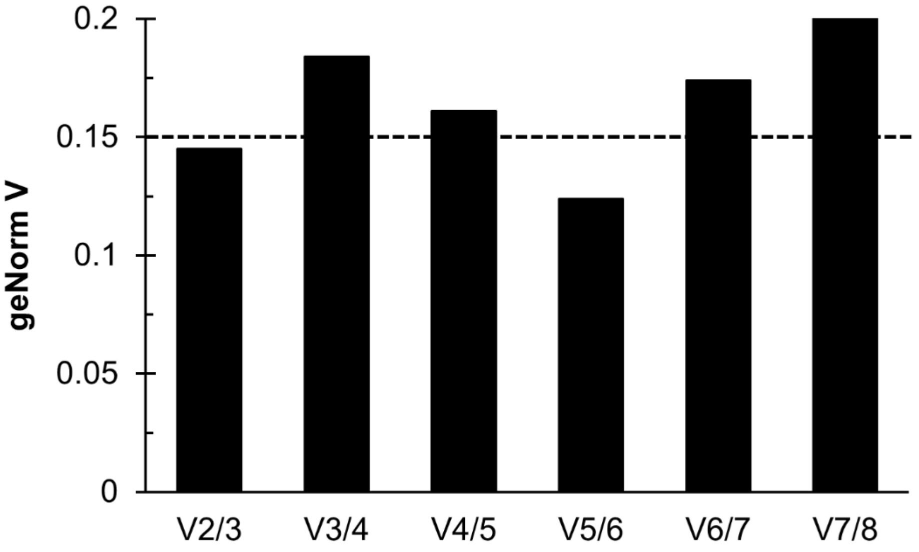

Supplementary Figure 3. Minimum number of reference genes (RGs) with rRNA removed. The pair-wise variation Vn/Vn+1, where n represents the number of RGs used in the normalization factor (NF), was calculated by geNorm to determine the minimum number of RGs required for normalization. A geNorm V value below 0.15 signifies that no additional benefit is gained from increasing the number of reference genes from n to n+1. The dashed line represents a V value of 0.15.

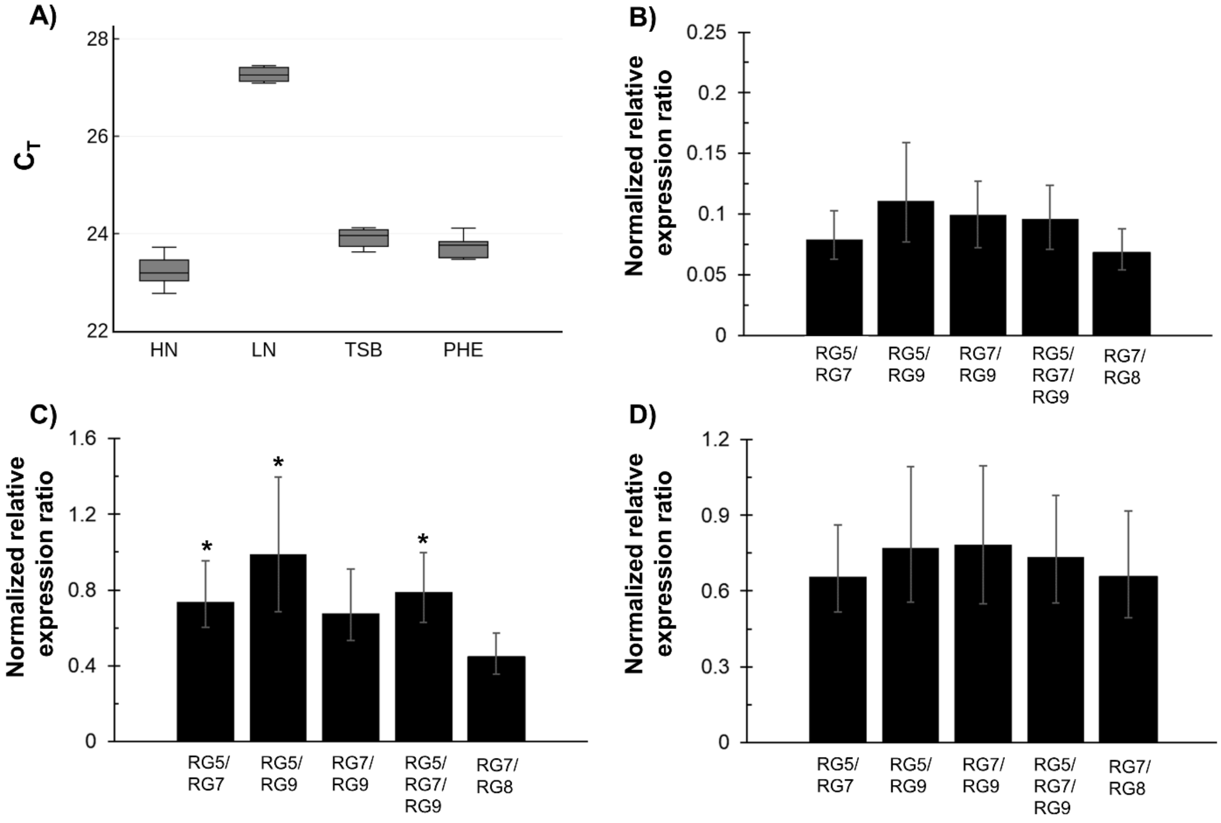

Supplementary Figure 4. Effect of reference gene choice on RT-qPCR normalization with rRNA candidates removed. **A)** Box plots of averaged PD630_RS15810 expression data (C_T) for all four growth conditions (HN, LN, TSB, and PHE). Each gray box represents the first through third quartiles, the solid black line represents the median, and the whiskers represent the minimum and maximum values. **B-D)** The normalized relative expression ratio of PD630_LPD05540 going from HN to either LN (B), TSB (C), or PHE (D). The expression data was normalized with either RG5/RG7, RG5/RG9, RG7/RG9, RG5/RG7/RG9, or RG7/RG8 using REST 2009. Error bars represent the 95% confidence interval (CI) as calculated by REST 2009. Stars indicate that a 95% CI range falls outside of the 95% CI range of the RG7/RG8 normalized ratio.

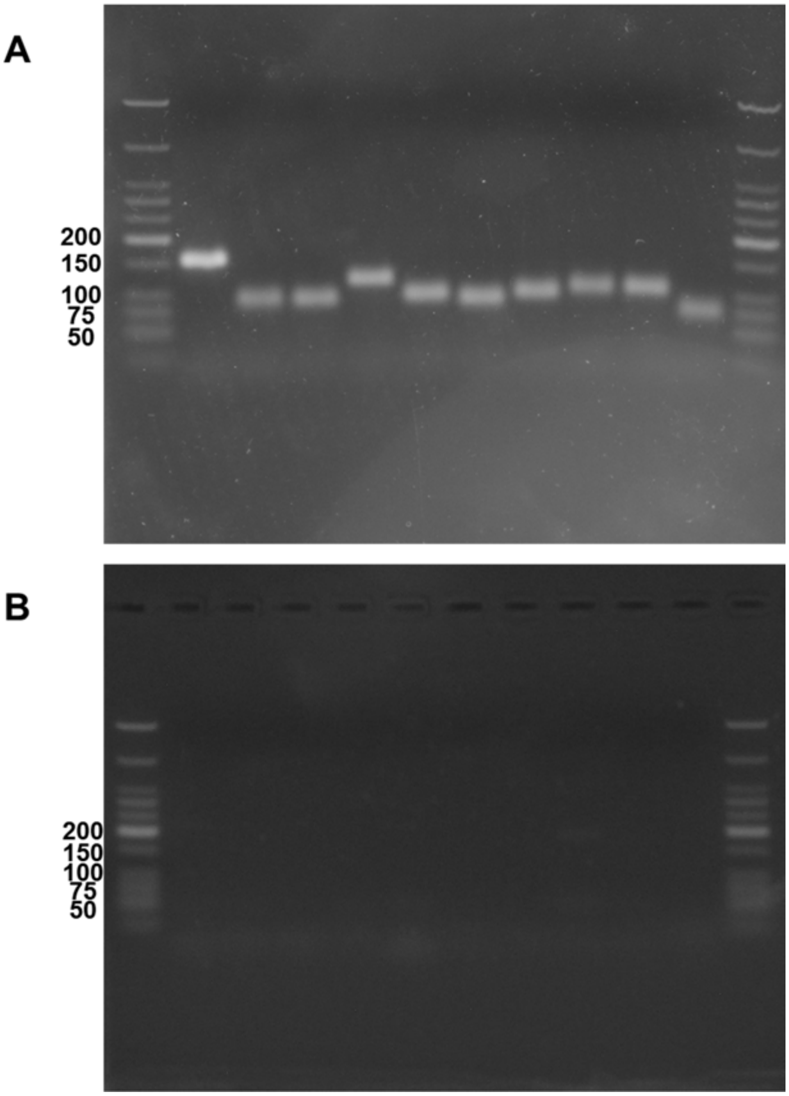

Supplementary Figure 5. The original version of electrophoresis gel images. **A)** The original non-edited, full length electrophoresis gel image showing single amplicon bands after RT-qPCR for each reference gene candidate (RG 1 to RG 10; left to right). **B)** The original non-edited, full length electrophoresis gel image confirming no bands for the negative template controls (NTCs) after RT-qPCR for each gene candidate (RG 1 to RG 10; left to right). The sizes for the nucleotide ladder are indicated to left of bands (50 to 200 bp). The size of each amplicon is denoted in Table 1.

RT-qPCR primer design criteria

Primer annealing temperature = ~60 ºC when using 125 nM primer (ThermoFisher Tm calculator)

Amplicon length = 70-160 bp

Try to have only one G/C at 3' end. No more than 2 G/Cs

Two or fewer consecutive G/C pairs in predicted hairpin structure (IDT OligoAnalyzer 3.1; https://www.idtdna.com/calc/analyzer); avoid binding on 3' end

Primer hairpin = $\Delta G > -2$ kcal/mol

Primer homodimer = $\Delta G > -7$ kcal/mol

Primer heterodimer = $\Delta G > -7$ kcal/mol
